# Investigating the quality and quantity of effluent in wastewater treatment plants of Iran: A case study of Tehran

**DOI:** 10.1016/j.mex.2018.07.015

**Published:** 2018-07-24

**Authors:** Mohammad Hadi Dehghani, Toktam Taleb Beydokhti

**Affiliations:** a Tehran University of Medical Sciences, School of Public Health, Department of Environmental Health Engineering, Tehran, Iran; b Tehran University of Medical Sciences, Center for Solid Waste Research (CSWR), Institute for Environmental Research (IER), Tehran, Iran

**Keywords:** Wastewater treatment plant, Effluent, Iran, Tehran

## Abstract

Control and monitoring of water sources from different types of contaminants especially collection and proper wastewater discharge is very important, in Iran which is considered as one of the dry regions in the world. Undesired quality of wastewater treatment plant’s (WWTPs) effluent causes many environmental problems.

In this research the parameters of Biochemical oxygen demand, Chemical oxygen demand, Total solids, Total suspended solids, Total dissolved solids, Potential of Hydrogen, Mixed liquor suspended solids, Mixed liquor volatile suspended solids, Sludge volume index, and temperature are examined based on the book of Standard Methods. The results of analyzing the above parameters which are related to eight WWTPs effluent out flow in Tehran, were analyzed by SPSS and Excel soft wares. The achieved data were compared to the standards of Iran’s Environmental protection agency. The results has shown that the parameters of Potential of Hydrogen, Chemical oxygen demand, Biochemical oxygen demand, and Total suspended solids of WWTPs effluent in Tehran are in accordance with standards of Environmental protection agency which are respectively 97.5%, 95.8%, 96.1%, 68.8%.

WWTPs of **C** has also the best operation among other WWTPs. In this study, due to ethical considerations, the name of the WWTPs is not mentioned. The names of the WWTPs are marked with **A–H**.

## Graphical abstract

**Figure:**
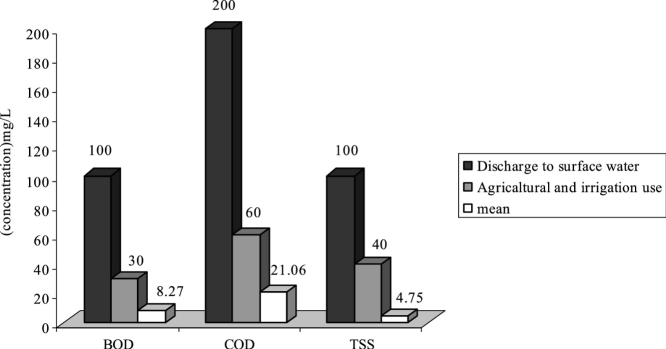

## Introduction

Protection of water sources from different types of contaminants and using the best methods of water treatment and water reuse should be considered more in Iran which is one of the dry regions in the world [1,2]. Untreated wastewater or wrong treatment will transfer the pathogens as well as polluting the sources of freshwater and will cause new limitations for the societies. Although fresh water is a revival source but can be severely polluted by human activities and became unusable for a long period of time. Increasing growth of the population has added the volume of the wastewater and the problems of discharging them in the environment will enter a more critical stage. This matter has caused problems for the existing wastewater treatment plants. We can refer to the overload that decrease the operation of the wastewater treatment plants and decline the quality of effluent outflow [[2], [3], [4]]. Above all mentioned cases, the important thing is its effect on human’s health which is as a result of dispersed bacteria and pathogenic viruses. Some of the wastes will create turbidity and color of water in receiving waters and display an obscene and indecent manifestation [[3], [4], [5]]. However in case of wastewater treatment, the problems of environmental pollutions such as sudden decrease of oxygen at the discharge point of wastewater treatment, Eutrophication, foam creation and death of hydras will be controllable [5,6].

This research is willing to study the quality and quantity of the effluent of wastewater treatment plants, according to chemical and physical parameters of effluents in different parts of Tehran and wants to compare it with discharge standards of the environmental protection agency of Iran.

## Materials and methods

There are eight wastewater treatment plants (WWTPs) in Tehran that their specifications are shown in Table 1. In order to assess the quality of discharged wastewater treatment plants to the environment, there are standards compiled in Iran and in the world. In these standards, the risks of different usage of effluent are taken into consideration. In this research the standards of Iran’s environmental protection agency is used. The most important chemical and physical parameters to study the WWTPs of **A–H** were as follows: Biochemical oxygen demand (BOD), Chemical oxygen demand (COD), Total solids (TS), Total suspended solids (TSS), Total dissolved solids (TDS), pH, Mixed liquor suspended solids (MLSS), Mixed liquor volatile suspended solids (MLVSS), Sludge volume index (SVI), and temperature. Spot sampling in the mentioned WWTPs were taken between 9 until 11 a.m., when the inputs to the WWTPs were in maximum amount. The special manner of sampling and the method of tests were taken according to the book of Standard Methods for the Examination of Water and Wastewater [7]. In order to do statistical analysis of the data, SPSS and Excel were used.

**Table 1:** Tehran’s wastewater treatment plants with activated sludge process.

NO.	Specification	H	G	F	E	D	C	B	A
1	Type of process	Extended aeration	Contact stabilization	Extended aeration	Deep aeration	Extended aeration	Extended aeration	Surface aeration	Extended aeration
2	Design population (Capital)	2000	60000	30000	12000	100000	85000	40000	80000
3	Entrance average discharge (design) m^3^/h	12	200	200	62.5	1000	1260	170	330
4	Entrance maximum discharge (design) m^3^/h	36	378	450	187.5	1875	2520	340	500
5	Entrance average discharge (present) m^3^/h	50	290	130	155	1250	720	425	560
6	Entrance maximum discharge (present) m^3^/h	100	350	270	380	1875	1440	625	600

## Results and discussion

**Fig. 1:**
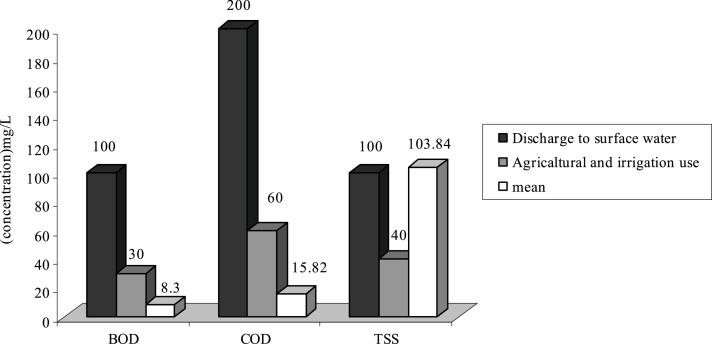
Comparison of average quantity of pollutants with standard in H WWTP.

**Fig. 2:**
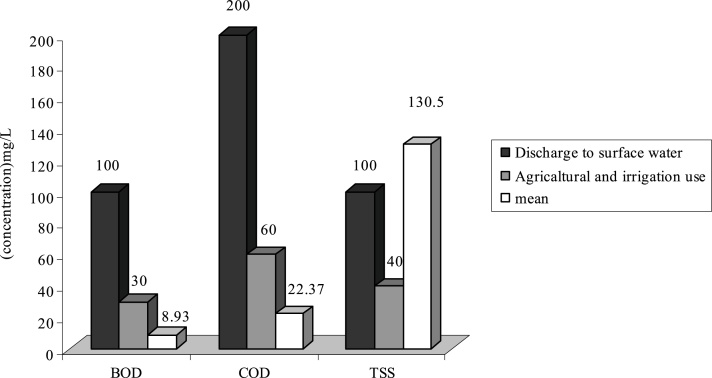
Comparison of average quantity of pollutants with standard in F WWTP.

**Fig. 3:**
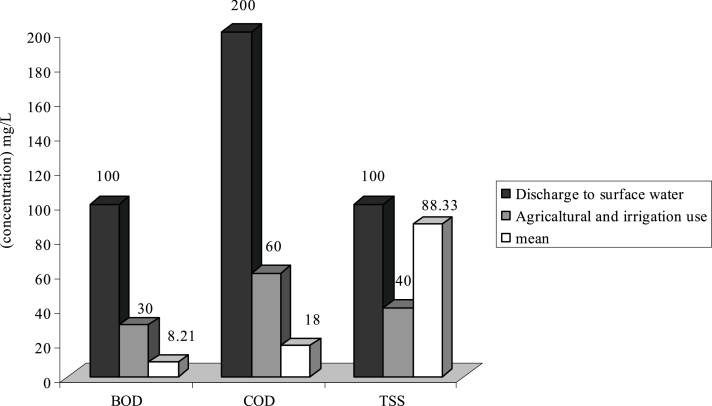
Comparison of average quantity of pollutants with standard in E WWTP.

**Fig. 4:**
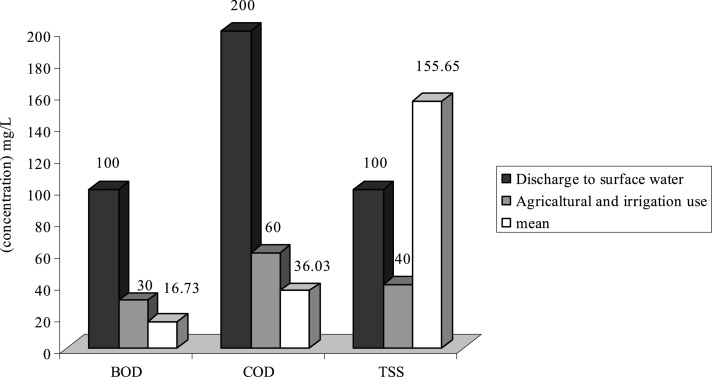
Comparison of average quantity of pollutants with standard in G WWTP.

**Fig. 5:**
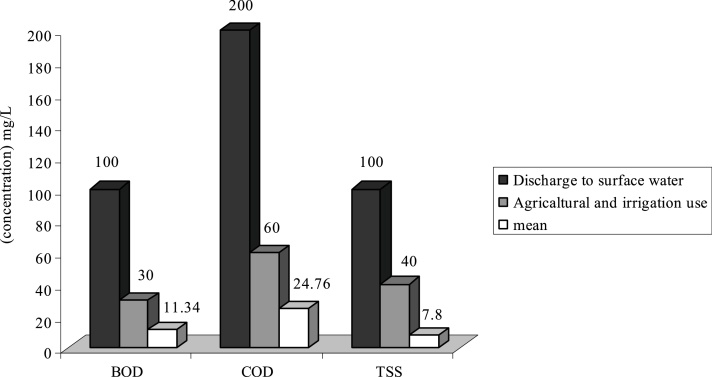
Comparison of average quantity of pollutants with standard in D WWTP.

**Fig. 6:**
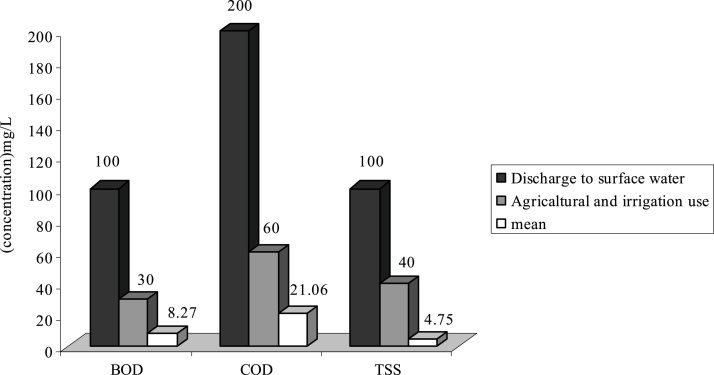
Comparison of average quantity of pollutants with standard in C WWTP.

**Fig. 7:**
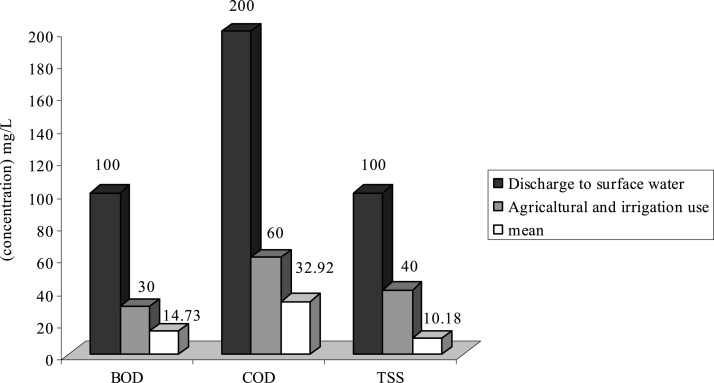
Comparison of average quantity of pollutants with standard in B WWTP.

**Fig. 8:**
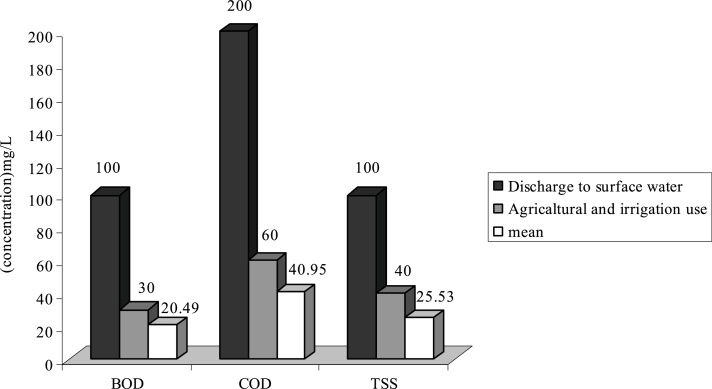
Comparison of average quantity of pollutants with standard in A WWTP.

In this research, the average of parameters that are studied according to the standards of Iran’s Environmental protection agency (Fig. 1, Fig. 2, Fig. 3, Fig. 4, Fig. 5, Fig. 6, Fig. 7, Fig. 8). As it is shown on Table 2 the highest percentage of BOD of standard output refers to treatment plants of **F, E** and **C** with 100% output standard and the lowest BOD of standard output refers to wastewater treatment plant of **A** with 78.3% output. However in all of the treatment plants we come to this conclusion that BOD of standards outputs was 96.1% which can be acceptable. According to the given data in Table 3, it can be seen that the highest percentage of COD of standard output refers to WWTP of **E** and **C** which are 100% and the lowest percentage of COD of standard output refers to WWTP of **A** with 84.4% and **G** with 90.9%. We can also conclude that the measurement of COD of standard output was 95.8% and only 4.2% of COD were non-standard.

**Table 2:** Frequency distribution of outlet standard and non-standard BOD for treatment plants.

Treatment plants	Standard		Non-standard	
	Number	percent	Number	percent
H	89	98.9	1	1.1
F	44	100	0	0
E	29	100	0	0
G	28	96.6	1	3.4
D	52	96.3	2	3.7
C	45	100	0	0
B	47	97.9	1	2.1
A	36	78.3	10	21.7
Sum	370	96.1	15	3.9

**Table 3:** Frequency distribution of outlet standard and non-standard COD for treatment plants.

Treatment plants	Standard		Non-standard	
	Number	percent	Number	percent
H	109	98.2	2	1.8
F	58	98.3	1	1.7
E	40	100	0	0
G	40	90.9	4	9.1
D	72	97.3	2	2.7
C	64	100	0	0
B	68	95.8	3	4.2
A	54	84.4	10	15.6
Sum	505	95.8	22	4.2

As shown in Table 4, the highest percentage of standard output pH refers to treatment plant of **B–D** and **H** with 100% and the lowest percentage of standard output pH refers to WWTP of **F** with 85.7% and **E** with 92.3%. Measurement of output pH shows that the standard outputs of pH was 97.5% in which only 2.5% was not standard. Table 5 shows that the highest percentage of standard output TSS refers to WWTP of **C** and **B** with 100% and the lowest percentage of standard output TSS refers to WWTP of **G** with 13%, **F** with 25%, **H** with 25.6% and **E** with 41.7%. Measurement of output TSS shows that standard output of TSS is 68.8% and non-standard cases is 31.2%.

**Table 4:** Frequency distribution of outlet standard and non-standard pH for treatment plants.

Treatment plants	Standard		Non-standard	
	Number	percent	Number	percent
H	110	100	0	0
F	48	85.7	8	14.3
E	36	92.3	3	7.7
G	43	97.7	1	2.3
D	75	100	0	0
C	65	100	0	0
B	72	100	0	0
A	63	98.4	1	1.6
Sum	512	97.5	13	2.5

**Table 5:** Frequency distribution of outlet standard and non-standard TSS for treatment plants.

Treatment plants	Standard		Non-standard	
	Number	percent	Number	percent
H	23	25.6	67	74.4
F	10	25	30	75
E	10	41.7	14	58.3
G	3	13	20	87
D	73	97.3	2	2.7
C	64	100	0	0
B	72	100	0	0
A	56	87.5	8	12.5
Sum	311	68.8	141	31.2

Other parameters of measurement at the input and output of WWTP consists of temperature, Total solids and Total liquid solids. Statistical analysis of the data shows that the highest average of temperature at the input of WWTPs of Tehran refer to **G** (25.39 ± 4.44) and the lowest average at the input of the WWTP refers to **C** (22.4 ± 3.55) Table 6. The most and the least average temperature of outputs of WWTPs respectively refers to **G** (25.47 ± 4.74) and **D** (21.31 ± 3.56) (Table 7). One of the other measured parameter is TS that its most and least average respectively refers to inputs of WWTP in **B** (1209.85 ± 203.24) and **H** (442.29 ± 144.66) (Table 6). The most and the least average can also be seen respectively at the output of WWTP in **A** (778.68 ± 75.83) and **H** (299.16 ± 123.67) (Table 7). The highest average of measured TDS at the input and output of WWTPs are respectively related to WWTP of **B** (899.07 ± 201.87) and **A** (753.31 ± 72.52) (Table 6, Table 7) and the lowest average of TDS is related to WWTP of **H** which is respectively (190.4 ± 104.11) and (197.34 ± 86.30) (Table 6, Table 7).

**Table 6:** Comparison of average quantity for other measured parameters into treatment plants.

Treatment plants	Temperature			Total Solids		
	Mean	Std.	Mean	Std.	Mean	Std.
		Deviation		Deviation		Deviation
H	23.57	5.28	442.29	144.66	190.4	104.11
F	24.66	5.38	553.27	145.48	262.25	133.04
E	22.42	6.43	582.75	195.57	305.45	178.90
G	25.39	4.44	629.04	191.69	325.83	158.85
D	23.99	2.61	684.80	95.20	479.24	104.36
C	22.40	3.55	652.72	118.41	488.34	113.82
B	23.45	4.15	1209.85	203.24	899.07	201.87
A	22.96	4.48	1086.82	212.22	763.01	176.85

**Table 7:** Comparison of average quantity for other measured parameters out of treatment plants.

Treatment plants	Temperature		Total Solids		Total Dissolved Solids	
	Mean	Std. Deviation	Mean	Std. Deviation	Mean	Std. Deviation
H	22.26	5.16	299.16	123.67	197.34	86.30
F	21.51	5.59	387.50	140.14	259.74	150.14
E	21.46	6.55	395.95	145.85	320	170.21
G	25.47	4.74	426.38	143.68	314.78	135.54
D	21.31	3.56	448.10	65.44	440.18	59.23
C	21.40	4.06	460.98	52.12	456	52.36
B	21.69	4.47	720.30	99.32	710.15	98.57
A	21.46	5.08	778.68	75.83	753.31	72.52

In this research, the parameters of aerated tanks in the specified WWTPs were also tested and analyzed (Table 8). The parameters of aerated tanks consisted of Sludge volume index (SVI), Mixed liquor suspended solids (MLSS) and mixed liquor volatile suspended solids (MLVSS) which are important in different conditions of operation in controlling activated sludge for protecting the operation of treatment. As it is shown in Table 8, the highest average of aerated tank with consideration of SVI, MLSS and MLVSS are respectively (405.08 ± 219.61) at **B** WWTP, (5980.32 ± 5432.89) and (4749.24 ± 4861.98) at **E** WWTP. The lowest average is also respectively (75.46 ± 73.67) at **G** WWTP, (1202.19 ± 412.66) and (1035.58 ± 347.67) at **D** WWTP. With a view to SVI, the average of data at the concerned treatment plants shows that, **G** is the only WWTP which can be placed at SVI = 50–150 (75.46) that states sludge with a good quality of settling.

**Table 8:** Comparison of average quantity for SVI, MLSS and MLVSS in treatment plants.

Treatment plants	SVI		MLSS		MLVSS	
	Mean	Std. Deviation	Mean	Std. Deviation	Mean	Std. Deviation
H	249.51	96.82	2591.23	879.02	1752.61	641.59
F	193.78	95.30	3004.41	1583.67	2342.47	1338.11
E	119.31	97.40	5980.32	5432.89	4749.24	4861.98
G	75.46	73.67	2857.61	2027.16	2257.01	1770.89
D	370.65	245.27	1202.19	412.66	1035.58	347.67
C	233.96	147.41	1771.74	825.25	1493.87	700.73
B	405.08	219.61	1806.42	771.87	1409.04	570.55
A	355.74	177.47	1526.66	774.07	1164.50	557.78

On the other part, in WWTPs where different methods of aeration is used (Table1), the amount of MLSS is also different, so that at extended aeration activated sludge process (**D, F, H**), the designing parameter for MLSS was equal to 1500–5000 mg/L at mentioned WWTPs. At WWTPs such as **G**, that use contact stabilization method, the designing parameter for MLSS is equal to 1000–3000 mg/L. In accordance with Table 8 the average of MLSS for this WWTP will also be at the same range as mentioned above.

As it is shown on Figs. 1, 2 and 4, the average of TSS at WWTPs of **H**, **F** and **G** is respectively 103.84 mg/L, 130.5 and 155.65 mg/L. Therefore the effluent of these WWTP is higher than standard in respect to disposal in surface water and agricultural consumptions and irrigation. Existence of other materials in output effluent of all WWTP is less than output standards of the country or they are just at that limit.

One of the common applicable methods is settle ability. To determine the rate of settle ability of activated sludge, the SVI parameter is used and it consists of occupied volume of one gram of suspended solids (MLSS). The index rate of sludge volume of 50–150 states that sludge had a good quality of settling. The higher sludge volume index, explains low quality of sludge settling [1,3]. In order to control the microorganisms’ activity in the activated sludge, controlling the MLVSS in wastewater treatment plants is done in cold weather condition, so that with more concentration of MLVSS in cold weather, the same treatment can be done as in hot weather with less concentration of MLVSS [14,15].

## Conclusions

According to the studies on mentioned wastewater treatment plants, the cause of high rate of SVI and low rate of MLSS is as follows:

Firstly, the percentage of return sludge was low and if it reaches 100–150 percent, the problem will be solved to large extent. Secondly, we can say that the SRT is low due to short intervals in discharging the sludge, where as in extended aeration systems, the produced sludge was insignificant and it should be disposed at long intervals. High SVI is due to low rate of SRT and MLSS and it will increase the suspended matters in effluent. On the other hand, the cause of high TSS at outlets of the wastewater treatment plants is having more than permissible input load, which decreases the residence time at aeration and settling tank and increases the TSS at outlet.

## Additional information section

The study held by Torabian [8] shows that the pH, BOD and TSS at WWTP of **H** was respectively (7.6, 26 mg/L and 23 mg/L) in **E** (7.6, 25 mg/L and 14.7 mg/L), in **G** (7.6, 30 mg/L and 19 mg/L), in **B** (7.7, 29 mg/L and 21 mg/L) and in **D** (7.5, 28 mg/L and 22 mg/L). All the mentioned parameters are at the standard level. The parameters of pH and BOD in this research are in concordance with studies of Torabian but the TSS parameter in Torabian’s study was standard, where as in this research for the reason of increase in population during the time and having overloads in input level and decrease in treatment time, the amount of TSS output at WWTPs of **E–H** appears higher than standard level. The study held at WWTPs of Tehran by Afshar [9], has come to this conclusion that the amount of pH, BOD,COD and TSS in WWTP of **H** was respectively (7.33, 11.7 mg/L, 24.8 mg/L and 27.7 mg/L), in **B** it was respectively (7.4, 20.3 mg/L, 37.5 mg/L and 21.4 mg/L) and in **E** it was respectively (7.24, 11.4 mg/L, 21.1 mg/L and 18 mg/L), that these results of pH, BOD and COD are the same with the results of this research and are at standard level. But unlike this research, the amount of TSS was reported standard in Afshar’s studies [9]. The amounts of BOD, COD and TSS at **D** WWTP was respectively reported 10 mg/L, 19 mg/L and 20 mg/L according to Gholami’s [10] study. The results in this research are in concordance with Gholami’s studies about **D** WWTP. The presented report by Dastmalchi [6] showed that the amount of pH, BOD and TSS in **H** WWTP was respectively 7.32, 24.83 mg/L and 18 mg/L that is in accordance with the results of this research on pH and BOD. But in contrary to this research, the amount of TSS was reported standard in Dastmalchi’s study [6]. According to Nouri sepehr’s study [11] at WWTP of **H**, the amount of pH, BOD, COD, and TSS were respectively reported 7.27, 15 mg/L, 18 mg/L and 40 mg/L. The results of his study are similar to the results of this research on pH, BOD and COD, but unlike this survey, the amount of TSS was reported standard in Nouri sepehr’s study [11].

A study by Joan Garcia [12] at WWTP of Cervera reported the amounts of BOD, COD and TSS as follows: 5 mg/L, 71 mg/L and 10 mg/L. All the mentioned parameters are at standard levels which are similar to the results of this research on parameters such as BOD and COD at wastewater treatment plants of Tehran. The results of his research on TSS are similar to the results of research on WWTP of **B–D**. The study held by FAO [13] showed that the amount of COD WWTP of Santa Rosa in Laguna (California) was 27 mg/L. The amount of pH, BOD and TSS in WWTP of Montecito Sanitary District was respectively 7.6, 11 mg/L and 13 mg/L which is in concordance with the results of this research on parameters of BOD and COD in WWTPs of Tehran. The results of above research on TSS parameter are similar to the obtained results of this research at WWTPs of **B–D**.

According to Nouri sepehr’s [11] study at WWTP of **H**, the amount of SVI, MLSS and MLVSS were respectively reported 290 ml/g, 1535 mg/L and 1230 mg/L. The results of his study are similar to the results of this research on SVI. But the amount of parameters of MLSS and MLVSS in Nouri Sepehr’s [11] surveys are lower than the one obtained in this research. The result of studies by FarzadKia [14] which were done at wastewater treatment plants of Tehran shows that the amount of SVI at WWTP of **B** was 109 mL/g, at **E** it was 135 mL/g, at **D** it was 128 mL/g and at **H** WWTP it was 148.25 mL/g. The amount of SVI at WWTP of **E** is similar to the one obtained in this research, but the amount of SVI in other WWTP are lower than the one obtained in this research.

The presented report by Gholami [10] on WWTP of **D** shows that the amounts of SVI, MLSS and MLVSS were respectively 147 mL/g, 6020 mg/L and 4751 mg/L where his results are not similar to the results of this research. According to studies of Rezaiyan [5] at WWTP of **D**, the amount of SVI and MLSS are respectively (109.1 ± 10.2) ml/g and (4692 ± 532.8) mg/L, where the obtained results in this study are not in concordance with those that he obtained. MLSS and MLVSS are the usual parameters used in controlling the activated sludge process. The controlling strategy of protecting the proper concentration of MLSS in the aeration reservoir or protecting the desirable thickness of sludge crust is based on the final clarifiers.
